# Adaptation of the molecular bacterial load assay for real-time quantification of *Mycobacterium tuberculosis* in the clinical routine laboratory

**DOI:** 10.1128/spectrum.01018-26

**Published:** 2026-08-18

**Authors:** B. Andersson, V. Kholod, M. Jonsson Nordvall, C. Braian, R. Kumar, K. Niward, T. Schön

**Affiliations:** 1 Department of Biomedical and Clinical Sciences, Division of Inflammation and Infection, Linköping University 568027, Linköping, Sweden; 2 Department of Clinical Microbiology, Linköping University Hospital 56750, Linköping, Sweden; 3 Department of Infectious Diseases, Linköping University Hospital, Linköping University 4566, Linköping, Sweden; 4 Department of Infectious Diseases, Kalmar County Hospital, Linköping University 4566, Kalmar, Sweden; Weill Cornell Medicine, New York, New York, USA; Gombe State University, Gombe State, Nigeria; University of St Andrews, St Andrews, Scotland, United Kingdom

**Keywords:** TB-MBLA, molecular bacterial load assay, *Mycobacterium tuberculosis*, heat inactivation, automated RNA extraction

## Abstract

Rapid quantification of *Mycobacterium tuberculosis* (Mtb) is important for monitoring bacterial load during treatment and in clinical studies. The molecular bacterial load assay detecting Mtb 16S ribosomal RNA (TB-MBLA) can quantify mycobacterial load within hours. As the current TB-MBLA protocol includes highly toxic chemicals and manual RNA extraction, we aimed to optimize TB-MBLA for routine clinical laboratories. We compared several strategies to inactivate Mtb in sputum samples, which would facilitate analysis outside a BSL3 laboratory. Heating at 80°C for 20 min was the most efficient inactivation strategy, whereas GTC-DTT and 1 M NaOH failed to inactivate Mtb sufficiently. We used Mtb-inoculated sputum samples to compare manual phenol-chloroform RNA extraction, as recommended by the TB-MBLA protocol, to an automated magnetic bead-based extraction. Automated RNA extraction reduced total analysis time by 2 h and performed equally to phenol-chloroform extraction, with a detection limit of close to 100 CFU in 10-fold dilution series (mean Ct value: 36.0 ± 1.4 automated versus 33.5 ± 3.6 manual, *n* = 6). Freezing samples in lysis buffer up to 14 days did not affect TB-MBLA performance (mean Ct value: 22.6 ± 0.3 fresh versus 22.6 ± 0.6 frozen, *n* = 6). The final TB-MBLA protocol quantified bacterial load up to 11 days faster than time-to-positive culture (TTP) (mean TTP: 11 days, 2–24 days, *n* = 9) in sputum from patients with pulmonary TB. We adapted the TB-MBLA method for faster throughput in a routine clinical laboratory by using inactivation at 80°C for 20 min, followed by automated RNA extraction, achieving equal sensitivity compared to the original TB-MBLA method, and avoiding manual extraction and toxic reagents.

**IMPORTANCE:**

The current tuberculosis molecular bacterial load assay (TB-MBLA) protocol relies on toxic chemicals and manual RNA extraction, making the procedure both time‑consuming and labor‑intensive. We optimized and validated a faster TB-MBLA strategy, which can be adapted for routine clinical laboratories, including inactivation by heating, to facilitate implementation in settings without a BSL3 laboratory.

## INTRODUCTION

Tuberculosis (TB) remains a leading cause of morbidity and mortality worldwide, despite the availability of effective treatment (1). A remaining challenge in TB management today is the lack of reliable tools to monitor microbiological treatment response in real time. Such biomarkers for monitoring bacterial load are critical for guiding clinical decisions, preventing unnecessary treatment prolongation, and accelerating the development of new shorter regimens in clinical trials (2).

Conventional methods for monitoring the treatment response are inadequate for TB. Sputum smear microscopy, although inexpensive and rapid, cannot distinguish viable from dead *Mycobacterium tuberculosis* (Mtb) (3). Molecular assays, such as Xpert MTB/RIF Ultra, have improved initial diagnosis and rifampicin resistance detection (1) but, similarly to microscopy, it detects Mtb long after bacterial killing. Thus, other methods are needed for monitoring bacterial load after treatment initiation. Mycobacterial culture is considered the gold standard for detecting viable bacteria but usually takes at least 2–3 weeks to yield positive results (4), and is resource demanding with a strict demand for a BSL3 laboratory, making it unsuitable for real-time decisions on bacterial load.

The tuberculosis molecular bacterial load assay (TB-MBLA) is an RNA-based real-time reverse transcriptase quantitative PCR (RT-qPCR) method that quantifies viable Mtb by measuring 16S ribosomal RNA (rRNA) (5, 6). Unlike messenger RNA, 16S rRNA is highly abundant and relatively stable, yet declines as treatment progresses, making TB-MBLA a sensitive tool for monitoring bacterial load and the microbiological response to treatment (7). Studies comparing TB-MBLA and time-to-positive culture (TTP) have demonstrated moderate to strong correlation between these methods (ρ = 0.5–0.73; Spearman) (7–10). In a prospective multicenter study of 210 patients treated for pulmonary TB, the rate of conversion to sputum negative was 3.3 times slower than estimated with Xpert MTB/RIF Ultra (7). TB-MBLA provides results within 24 h compared to culture (4, 7, 10), and it was acknowledged by WHO in 2018 for monitoring treatment response, recognizing its potential as a surrogate marker for bacterial viability (11). The current TB-MBLA protocol requires labor-intensive manual RNA extraction (6), substantially limiting its scalability for routine clinical laboratories and large-scale trials. This may partly explain why no studies to date have reported real-time measurements of TB-MBLA. Additionally, there is a lack of studies evaluating a suitable procedure for and the impact of storing the samples before analysis. Moreover, the current TB-MBLA protocol relies on toxic chemicals, such as beta-mercaptoethanol, phenol, and chloroform. To meet the urgent need for a real-time biomarker for the treatment response adapted for the routine clinical laboratory, our aim was to optimize the currently used TB-MBLA, avoiding manual extraction and incorporating an efficient Mtb inactivation step to facilitate analysis outside of BSL3 laboratory.

## MATERIALS AND METHODS

### Preparation of sputum inoculated with Mtb

Mtb strain H37Rv ATCC 27292 was thawed and cultured in Middlebrook 7H9 broth supplemented with Tween-80 and 10% ADC (Becton Dickinson, Franklin Lakes, NJ, USA) for 2 to 3 weeks at 37°C. Bacterial culture was reinoculated into fresh broth 7 days prior to use in the experiments. The Mtb culture was twice washed in phosphate-buffered saline (PBS) + 0.05% Tween-80. A single-cell suspension of Mtb H37Rv in PBS was prepared by filtering the suspension through a 0.5 µm filter after the last washing step. The concentration of Mtb in PBS was determined by optical density at 600 nm as a function of CFU/mL according to a standard curve as previously described (12). Briefly, Mtb H37Rv was cultured to early log-phase, and CFUs were determined by plating the bacterial suspensions at four different OD values on three individual agar plates, which resulted in a standard curve range of 1.6–7.1 × 10^7^/mL (12). Excess sputum negative for Mtb from patients submitted to the routine Clinical Microbiological laboratory at Linköping University Hospital was pretreated with an equal amount of sputolysine (EMD Milipore Corp., Burlington, MA, USA) according to the protocol. As previously described for the routine Mtb culture conditions (13), we found no impact of sputolysine on Mtb viability (data not shown). Sputum was pooled from 50 to 100 donors, where 10 mL aliquots were stored at −80°C. Before use, sputum was thawed, and Mtb was added in a 1:10 ratio and vortexed for 30 s.

### Evaluation of Mtb inactivation in sputum samples

Inoculated sputum containing 6 × 10^6^ CFU/mL of Mtb was used to evaluate the inactivation protocols. The 4M GTC working solution was prepared and used as recommended in the TB-MBLA manual (Tuberculosis Molecular Bacterial Load Assay, SOP v.8 and 9, Vitalbacteria, St Andrews, Fife, UK). Briefly, 20 g GTC powder was dissolved in 12 mL RNase-free H_2_O during overnight incubation at 37°C on a shaker, and 4 mL 1 M Tris-HCl, pH 7.5 was added the following day. Beta-mercaptoethanol (1%) was replaced by 2 M DTT at 2% of the working solution volume, as previously described (14). Sputum inoculated with Mtb was mixed 1:4 with GTC-DTT working solution and incubated for 15 min. After washing once with PBS buffer, the suspension was inoculated to Löwenstein-Jensen (LJ) media. To evaluate the acetic acid inactivation strategy, inoculated sputum was treated with 8% acetic acid (final concentration) for 1 h at RT (15), washed once in PBS, and inoculated into LJ media. To evaluate NaOH treatment, inoculated sputum was mixed 1:1 with 1 M NaOH, incubated for 15 min at RT, washed once in PBS, and then inoculated into LJ media. To evaluate heat inactivation, 1 mL of inoculated sputum was added to 1.5 mL sterile screw-cap microtubes (Sarstedt, Nümbrecht, Germany) and incubated in a water bath at 80°C for 20 min. Samples were allowed to cool for at least 5 min and then inoculated into LJ media and BACTEC 960 MGIT, where samples were incubated for 6 weeks.

### Sample preparation for TB-MBLA

We followed the sample preparation procedure as recommended in the TB-MBLA manual (Vitalbacteria, St Andrews, Fife, UK) unless otherwise specified. Sputum samples containing Mtb, as specified for each extraction protocol, were handled outside the BSL3 laboratory following heat inactivation. To each sputum sample, 100 µL of extraction control provided in the TB-MBLA kit (Vitalbacteria, St Andrews, Fife, UK) was added (6, 16). Samples were centrifuged, and the supernatant was discarded while keeping the pellet intact. Pellets were resuspended in the lysis buffers described in the RNA extraction section below, transferred to Lysing Matrix B tubes containing 0.1 mm silica beads (MP Biomedicals, Irvine, CA, USA), and homogenized using a FastPrep bead-beater (MP Biomedicals, Irvine, CA, USA) for 40 s at 6.0 m/s, or alternatively using BeadBug Mini homogenizer D1030 (Benchmark, Sayreville, NJ, USA) at maximum speed for two bursts of 40 s cycles separated by a 40 s rest. Homogenized samples in Lysing Matrix B tubes were either stored at −80°C or processed immediately for RNA extraction. RNA concentration and purity were analyzed using Nanodrop One (Thermo Scientific, Thermo Fisher, Waltham, MA, USA).

### Manual phenol-chloroform extraction of RNA

The RNA was extracted using the FastRNA Pro Blue kit (MP Biomedicals, Irvine, CA, USA) from samples containing 1 mL sputum, as recommended by the TB-MBLA manual (Vitalbacteria, St Andrews, Fife, UK). Samples were lysed and homogenized in 950 µL RNA Pro Solution lysing buffer (MP Biomedicals, Irvine, CA, USA), followed by chloroform extraction. Samples were stored overnight at –20°C after the precipitation step with 100% ethanol, and purification continued the following day. The supernatant was removed from the pellets by pipetting prior to drying the RNA at 50°C. RNA was then rehydrated in 100 µL nuclease-free water and subjected to DNase treatment using the Invitrogen Turbo DNA-free kit (Invitrogen, Thermo Fisher, Waltham, MA, USA) according to the manufacturer’s instructions. The total RNA extraction time was approximately 2–3 h, including 1.5–2 h of hands-on time.

### Automated extraction of RNA with Maelstrom Switch8

The input sputum volume for the automated protocol was 250 µL. Samples were lysed and homogenized in 800 µL lysis buffer supplied in the Tissue RNA Extraction Kit (TANBead Nucleic Acid Extraction Kit, 6K2 series, Taiwan Advanced Nanotech, Taoyuan City, Taiwan). Equal volumes of lysate and isopropanol (500 µL each) were then combined and processed for RNA extraction on the Maelstrom Switch-8 automated magnetic-bead instrument (TANBead, Taiwan Advanced Nanotech, Taoyuan City, Taiwan) using the SW8-6K2 program. An aliquot of RNA eluate (20–60 µL) was subjected to DNase treatment directly after extraction, using the Invitrogen Turbo DNA-free kit (Invitrogen, Thermo Fisher, Waltham, MA, USA) according to the manufacturer’s instructions. The total RNA extraction time was approximately 1 h, including 10 min of hands-on time. A detailed protocol for the automated extraction method is provided as a Supplemental method.

### Reverse transcription and quantitative PCR

One-step RT-qPCR was performed using the Vitalbacteria TB-MBLA kit as recommended by the manufacturer (Vitalbacteria, St Andrews, Fife, UK). Reaction components for both the RT^+^ and RT^−^ master mixes with and without reverse transcriptase enzyme, respectively, were pre-mixed after kit arrival and stored at –20°C. One reaction contained 4 µL of sample and 16 µL of master mix. Samples were analyzed in duplicate in the RT^+^ reaction and in a single reaction in the RT^−^ reaction on a Microamp Fast Optical 96-well plate in the QuantStudio 7 Flex system (Applied Biosystems, Thermo Fisher, Waltham, MA, USA) using FAM and VIC channels for Mtb 16S rRNA and extraction control, respectively. An extraction control inoculated at 100 µL in each sample was used for normalization to compensate for the variation in RNA recovery. Statistical differences were assessed using two-way ANOVA with Šidák’s multiple comparisons test in GraphPad Prism version 10.0.2. Data are presented as mean ± SD, and a *P* value < 0.05 was considered statistically significant. The determination of the limit of detection (LOD) and limit of quantification (LOQ) was performed by replicate dilutions of a standard curve from 10^1^ to 10^4^ CFU/mL in sputum. The intra- and inter-assay variability was determined by the coefficient of variation (CV%) with a target below 10%.

### Conversion of Ct values to CFU/mL

A 100-fold dilution series of Mtb was inoculated into negative sputum in duplicate, starting at 1 × 10^7^ CFU/mL sputum. TB-MBLA was performed using the adapted TB-MBLA protocol incorporating automated RNA extraction. A standard curve correlating Ct values with the concentration of Mtb-inoculated sputum samples (CFU/mL) was generated by linear regression in GraphPad Prism version 10.0.2.

### TTP determination

For comparison to TB-MBLA of clinical samples, a 250 µL aliquot of the sputum sample was collected for TB-MBLA analysis, and 500 µL of the same sputum sample was processed for TTP analysis according to the local standard procedure. Sputum samples were decontaminated with sodium lauryl sulfate 1:1, according to clinical routine. Samples were neutralized with 0.09% sulfuric acid, washed in phosphate buffer, and inoculated into BACTEC MGIT mycobacterial growth indicator tubes (Becton Dickinson, Franklin Lakes, NJ, USA) together with MGIT PANTA growth supplement (BD). Tubes were incubated in BACTEC MGIT 960 (Becton Dickinson, Franklin Lakes, NJ, USA) at 37°C until positive or terminated after 42 days if no growth was detected.

## RESULTS

### Inhibition of *Mycobacterium tuberculosis* growth in sputum samples

Replacing beta-mercaptoethanol with treatment for 15 min with either a GTC working solution containing 2% 2 M DTT or 1 M NaOH resulted in clearly visible growth of 6 × 10^6^ CFU Mtb/mL sputum in all experiments (*n* = 3). In contrast, both incubation at 80°C for 20 min (*n* = 3) and treatment with 8% acetic acid for 1 h (*n* = 2) completely inhibited growth on LJ of the same amount of Mtb in inoculated-sputum samples. Heat inactivation at 80°C for 20 min did not show growth on MGIT either and was selected for subsequent experiments, as it allowed downstream handling of samples outside the BSL3 laboratory without the need for additional chemicals. In control experiments, using *M. bovis BCG* as a representative of the Mtb complex, we found no difference in TB-MBLA Ct-values from inactivation at 80°C for 20 min (Table S1).

### Comparison of automated and manual phenol-chloroform protocol

We evaluated whether automated RNA extraction could replace manual phenol-chloroform extraction to reduce hands-on time and labor. Maelstrom Switch8 (TANBead) instrument was selected during initial optimization with *Mycobacterium avium* since it resulted in significantly lower Ct values and a higher sensitivity compared to extraction with Qiagen EZ2 with EZ1&2 RNA Tissue Mini Kit, which failed to detect the lowest concentration tested (*n* = 2, Fig. S1).

**Fig 1:**
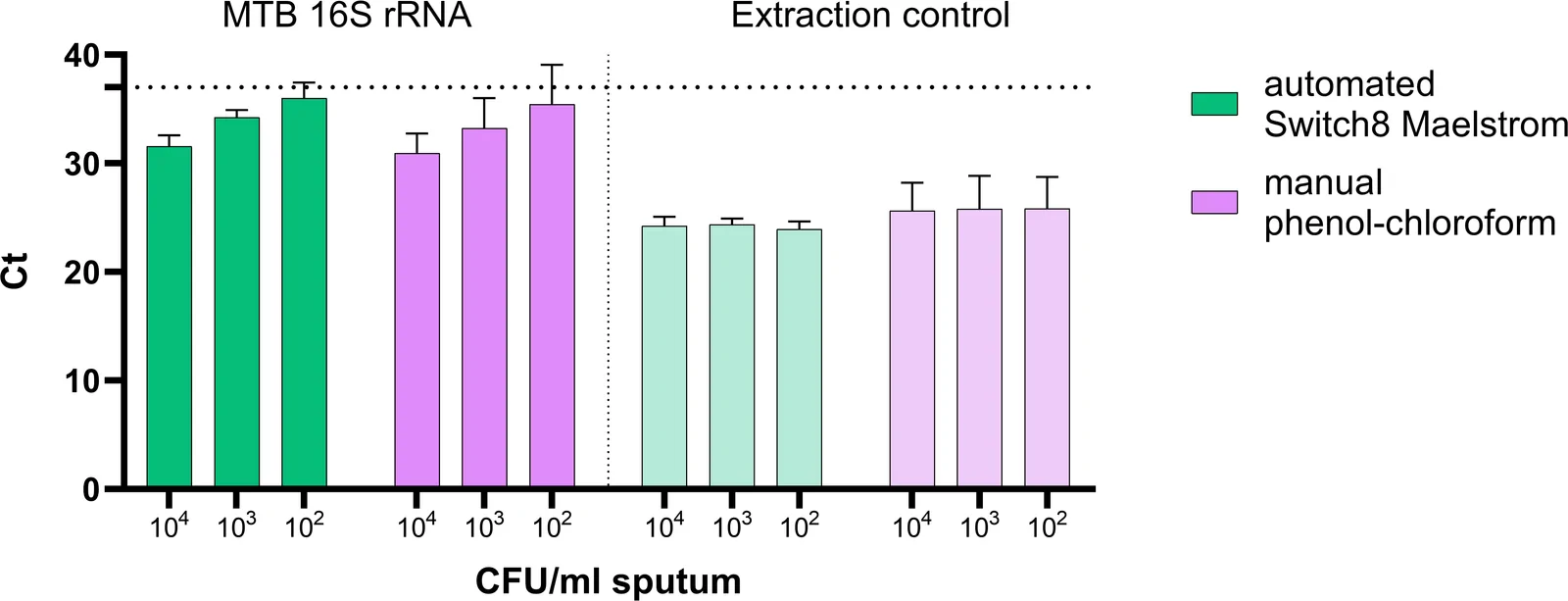
Comparison of manual phenol-chloroform and an automated magnetic bead-based RNA extraction protocol. Dilution series of Mtb at a 10-fold range were inoculated in sputum in duplicates. Samples were heat-inactivated at 80°C for 20 min and RNA extracted by the manual phenol-chloroform protocol using FastRNA Pro Blue kit and the automated protocol using TANBead Nucleic Acid Extraction Kit in parallel. An extraction control (100 µL/sample) was added to all samples prior to the extraction. RNA was diluted 10-fold, and Mtb 16S rRNA was quantified by TB-MBLA RT-qPCR. Data are presented as mean Ct values ± SD (*n* = 6), and the Ct values achieved by manual extraction were adjusted for a four times higher starting sputum volume (1 mL vs 250 uL for the automated protocol). There were no significant differences in Ct values between the manual and automated methods. The dotted line indicates the LOD at 37 cycles determined by the automated protocol using the QuantStudio 7 Flex system.

**Fig 2:**
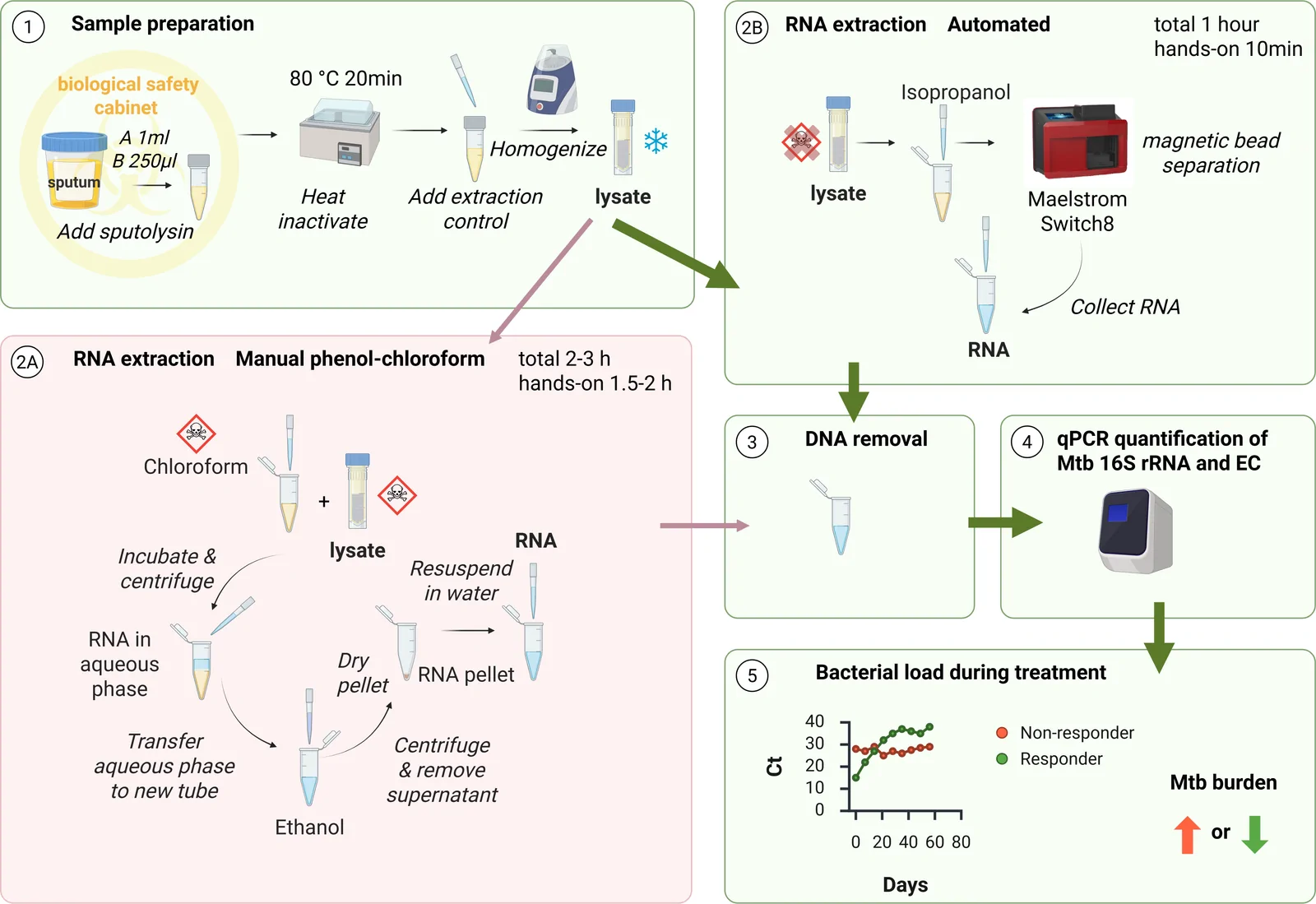
The adapted TB-MBLA workflow reduces the turnaround time by 1–2 h and decreases hands-on time by 1.5–2 h, thereby simplifying sample processing in routine laboratories. Sample preparation (1) includes heat inactivation at 80°C for 20 min, enabling subsequent handling outside the BSL3 laboratory. Homogenized samples can be stored at −80°C directly in Lysing Matrix tubes. The conventional manual phenol-chloroform RNA extraction method (2A; 2–3 h) is replaced by an automated RNA extraction using magnetic bead separation (2B; 1 h). RNA for both Mtb 16S rRNA and the extraction control is, after DNA removal (3), quantified by RT-qPCR (4) to monitor treatment response (5). The illustration was created using BioRender.

RNA was extracted from sputum samples inoculated with 10-fold serial dilutions of Mtb comparing manual and automated extraction protocols in parallel. There were no differences in RNA concentrations between protocols when concentrations obtained by the manual protocol were adjusted for the four times higher starting sputum volume, whereas RNA purity was slightly higher for the phenol-chloroform protocol (Table S2). In contrast to the automated extraction, RNA obtained by manual phenol-chloroform extraction did not consistently yield amplification in the TB-MBLA RT-qPCR unless 10-fold diluted (Table S3). When all RNA samples were 10 times diluted and the mean Ct values obtained using the manual extraction protocol were adjusted for the four times higher starting sputum volume, the mean Ct values of the automated protocol were comparable to and showed lower variation than manual phenol-chloroform extraction (Fig. 1; Table S4). There was no significant difference in mean Ct values for samples extracted by the automated protocol compared to mean Ct values for samples extracted by manual phenol-chloroform protocol (Fig. 1). The extraction control remained stable for both the automated and manual extraction methods (Fig. 1). The adapted protocol using automated RNA extraction reduced the turnaround time for the TB-MBLA by 1–2 h and hands-on time by 1.5–2 h (Fig. 2). The LOD was 100 CFU/mL sputum or a Ct cut-off at 37 which gave an amplification with a three-cycle difference in Ct-values compared to 1,000 CFU/mL sputum (Table S5). A Ct cut-off at 35 was determined as the LOQ, as there was consistent amplification at 100% of replicated samples (Table S5). The intra-assay variability (CV%) was 0.5% and the inter-assay variability 1.7%, which was well below the 10% target.

### Implementation of the adapted MBLA method in the routine laboratory

**Fig 3:**
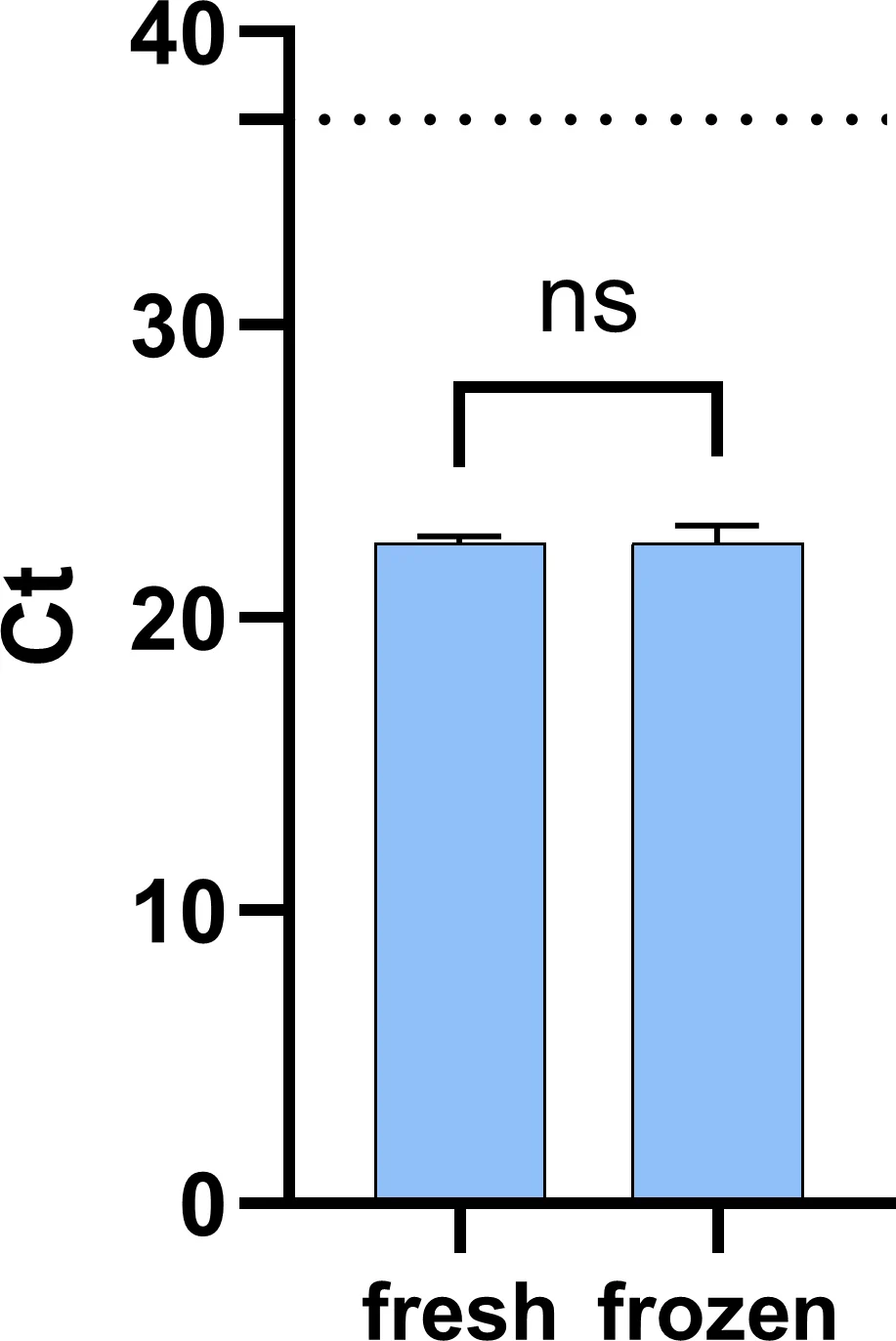
Effect of freezing on the RNA recovery. Samples containing 1 × 10^6^ CFU/mL sputum were prepared in duplicates, and RNA was extracted directly, or samples were stored at –80°C for 2 weeks. Mtb 16S rRNA was quantified by RT-qPCR following the automated RNA extraction protocol. Data are presented as mean Ct values, and significant differences between groups were calculated using a paired *t*-test (*n* = 6); non-significant difference is depicted as ns. The dotted line indicates the assay detection limit of 37 cycles.

**Fig 4:**
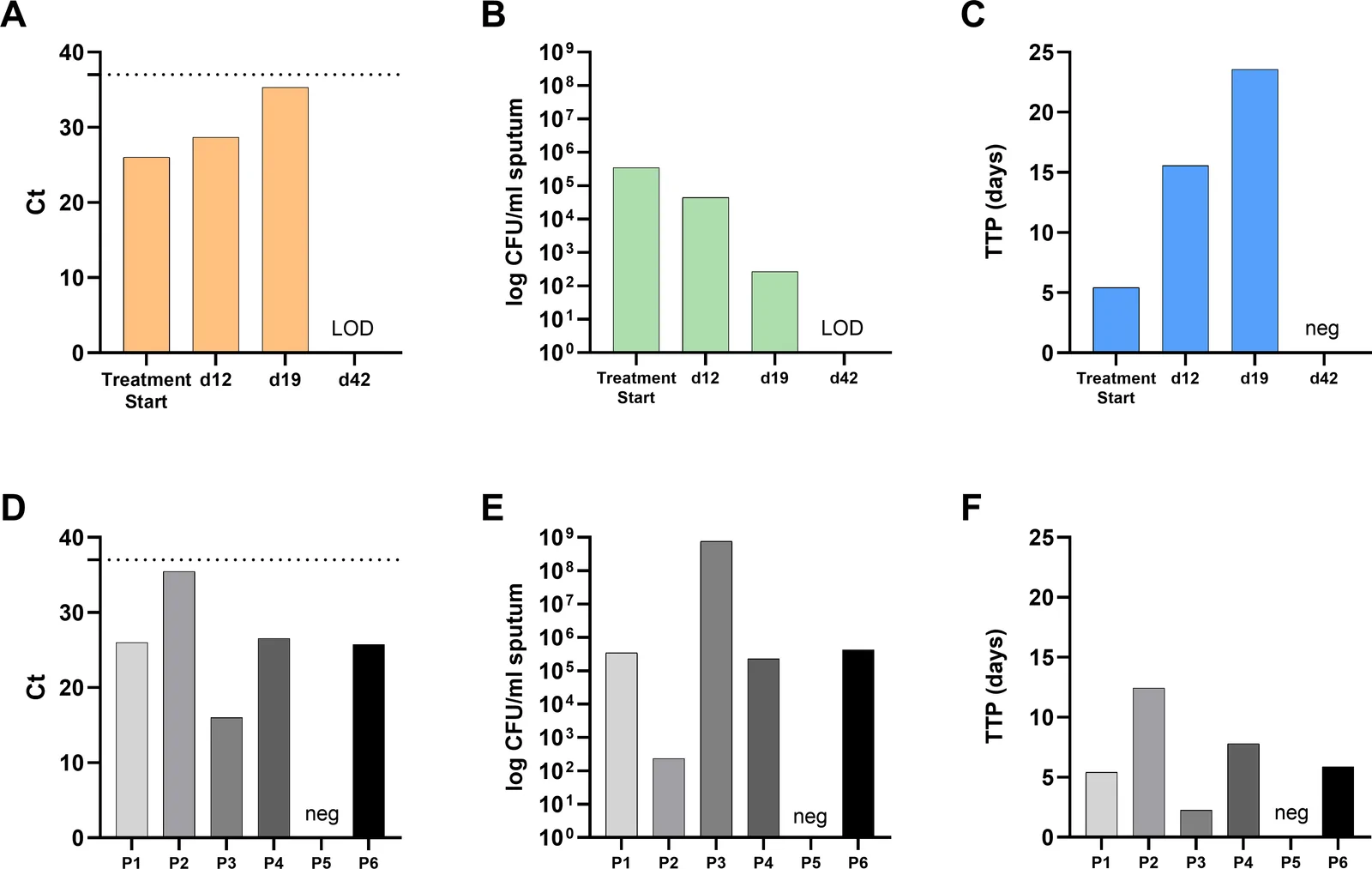
Monitoring the Mtb bacterial load and treatment response in clinical samples. Sputum aliquots (250 µL) were heat-inactivated at 80°C for 20 min, homogenized, and stored at −80°C. RNA was extracted using the automated protocol (Maelstrom Switch8) and quantified using TB-MBLA RT-qPCR. Samples were in parallel cultured in MGIT mycobacterial growth indicator tubes for TTP determination. The bacterial load in patient (P1) was followed for 6 weeks of treatment and expressed as Ct values (**A**), CFU/mL sputum (**B**), and TTP (**C**); neg indicates no growth detected after 42 days of culture, or no Ct value or amplification curve detected in qPCR. The difference in bacterial load between patients (P1–P4) at the start of treatment is expressed as Ct values (**D**), CFU/mL sputum (**E**), and TTP (**F**). The dotted line indicates the assay detection limit of 37 cycles.

In total, nine clinical patient samples from six patients (P1–P6; Table 1) were processed in a routine clinical microbiological laboratory using the adapted TB-MBLA protocol along with TTP determination by MGIT culture. After heat inactivation, samples for TB-MBLA were handled outside the BSL3 laboratory, homogenized using bead beating in a BSL2 cabinet, and stored at –80°C. Freezing the homogenized samples for 2 weeks in Lysing Matrix tubes did not affect performance, as confirmed using Mtb-inoculated sputum (Fig. 3). P1 was monitored for 6 weeks of tuberculosis treatment with first-line drugs. Both Ct values and TTP increased with the length of the treatment of P1 (Fig. 4A and C), corresponding to a decrease in bacterial load. The mycobacterial load was estimated from the standard curve (Fig. S2) to 3.4 × 10^5^ CFU/mL sputum at the treatment start, decreased to 4.4 × 10^4^ CFU/mL on treatment day 12 (Fig. 4B), and approached the detection limit for TB-MBLA on day 19 while detected culture positive with TTP of 23 days and 14 h. Sputum collected on day 42 was very close to the detection limit (Ct 36.9) in TB-MBLA (Fig. 4A) and was negative in TTP (Fig. 4C). We further compared the bacterial load in six patients (P1–P6) at the start of treatment (Fig. 4D). P3 showed the lowest Ct value (16.0), corresponding to 7.6 × 10⁸ CFU/mL sputum (Fig. 4D and E), and reached culture positivity in 2 days and 6 h (Fig. 4F). P2, with a Ct value near the detection limit (35.5), was determined culture positive after 12 days and 11 h, and P4, with a Ct value of 26.5, was determined culture positive after 7 days and 19 h (Fig. 4D and F). No Ct value or amplification curve was detected for P5, and the patient was also found culture negative (Table 1). All clinical samples from P1–P6 were negative in the control RT^−^ reaction as a control for DNA contamination, showing either no amplification signal or signal below the detection limit (data not shown). The results from TTP had an average response time of 11 days (2–24 days), whereas results from the TB-MBLA analysis could be received within 24 h after sample delivery.

**TABLE 1:** Baseline characteristics for patients analyzed by the automated TB-MBLA protocol^*a*^

Patient	Age	Sex	CXR cavity	Smear	Xpert	TTP	MBLA CFU/mL	Lineage	AST	Trx
1	61	M	Yes	3+	High	130	3.4 × 10^5^	4.5	S	HRZE
2	37	M	No	Neg	Low	299	2.3 × 10^2^	4.4.1.1	S	HRZE
3	14	F	Yes	3+	High	54	7.8 × 10^8^	2.2.1	S	HRZE
4	42	M	Yes	2+	Low	187	2.3 × 10^5^	4.2.2.1	S	HRZE
5	86	M	No	Neg	Neg	0	Neg	ND	ND	None
6	70	F	Yes	2+	High	141	4.3 × 10^5^	3	S	HRZE

^*a*^ CXR, chest X-ray; smear, smear grade (neg, 1+, 2+, or 3+); TTP, time to positive culture in BACTEC 960 MGIT in hours; AST, antimicrobial susceptibility testing for first-line drugs using whole-genome sequencing; HRZE, isoniazid, rifampicin, pyrazinamid, and ethambutol; Trx, treatment; ND, not determined.

## DISCUSSION

We developed and validated a faster TB-MBLA strategy adapted for routine clinical laboratories, including Mtb-inactivation by heating at 80°C and automated instead of manual RNA extraction, reducing hands-on time and the need for toxic chemicals. Additionally, we validated this strategy for batch sampling, enabling samples to be stored at −80°C while preserving TB-MBLA performance.

Our adapted TB-MBLA method achieved a 2 h shorter turnaround time and at least a 1.5 h reduction in hands-on time per sample while maintaining performance comparable to the current TB-MBLA protocol, which includes manual RNA extraction. Unlike the original protocol, which utilizes toxic organic solvents, such as phenol and chloroform for lysis, the automated protocol employs the TANBead lysis buffer based on the less toxic guanidine salt. In an effort to reduce exposure to hazardous reagents, we replaced the toxic reagent beta-mercaptoethanol with DTT in the GTC solution (14) for the initial inactivation of Mtb in the sputum samples and additionally evaluated 1 M NaOH, but neither of these alternatives sufficiently inactivated Mtb growth. Although the current TB-MBLA protocol considers inactivation with GTC-beta-mercaptoethanol safe for handling the samples outside the BSL3 laboratory (17), insufficient inactivation of Mtb by GTC-beta-mercaptoethanol or GTC-DTT has been reported (15). In contrast, both heat inactivation at 80°C for 20 min and treatment with 8% acetic acid reliably inhibited Mtb growth in our study, consistent with previous findings (6, 15, 17). We selected heat inactivation at 80°C for 20 min for the final protocol as it allowed easier handling of the samples during inactivation without the need for additional chemicals. Moreover, as heat inactivation did not require the addition of any reagents, the sample volume remained substantially unchanged, enabling more efficient pellet recovery using higher centrifugation forces and shorter spin times. Eliminating reagent preparation and addition of reagent also increased feasibility and reduced the risk of user error. Notably, heat inactivation had a low impact on Mtb quantification, as recovery from heat-inactivated samples remained close to 100% relative to the non-inactivated control (Table S1), in agreement with previous observations using the original TB-MBLA method (18). It should be noted that any inactivation protocol must be carefully validated under local conditions. Even if we did not detect any growth using heating to 80°C as an inactivation procedure for clinical samples in our routine laboratory for several years, we recommend handling samples in a BSL2 cabinet following heating while preparing samples for RNA extraction and during bead beating since low-level growth of Mtb has been reported (19–21). One of our goals was to facilitate the performance of the TB-MBLA method outside of the BSL3 laboratory for more widespread use and to avoid the requirement for special equipment in the BSL3 laboratory. There is also an alternative to perform the whole procedure within the BSL3 laboratory, but that requires all equipment for the assay to be present in the BSL3 laboratory. In our evaluation, we used both LJ and MGIT, reported previously to have a LOD of 10–100 and 1–10 CFU, respectively (22, 23), and no growth was found in any media following heat inactivation.

We compared the manual phenol-chloroform extraction to the automated extraction based on magnetic bead separation by evaluating inoculated sputum samples containing a dilution series of Mtb from 1 × 10⁴ to 1 × 10² CFU/mL. Ct values of samples processed by the automated protocol were generally slightly higher and closer to the detection limit than samples processed by the manual phenol-chloroform extraction. The clinical impact of this difference in the automatic extraction is likely minor, as the TB-MBLA is used for monitoring bacterial load and not for initial diagnosis, where the sensitivity for detection is optimized by methods such as Xpert MTB/RIF Ultra or culture. Also, the increase in Ct values following treatment against TB accurately reflected the reduction in Mtb concentration across the dilution series, and no significant differences in Ct values were observed between the two protocols. Moreover, RNA obtained using the manual protocol required a 10-fold dilution (Table S3), an effect that was not observed for RNA extracted with the automated protocol. The reason for this difference is unknown but may involve inhibition from carryover chemicals in the phenol-chloroform extraction on the RT-qPCR reaction. Ct values generated by the automated workflow showed lower variability compared with the manual method, indicating that automation provides more consistent results and is better suited for standardization. The main clinical application of TB-MBLA is not primary diagnosis but to monitor the bacterial load during the first weeks of treatment as an alternative to the much slower TTP method. The TB-MBLA only quantifies replicating bacteria and may miss dormant subpopulations. Furthermore, the CFU levels are transposed from a standard curve of Mtb grown *in vitro* as an approximate representative of rapidly dividing Mtb in human sputum samples. We found that the limit of detection by the automated extraction methods was 10² CFU/mL, which is very close to the LOD reported by the group developing the original TB-MBLA assay (5).

With the adapted TB-MBLA, we successfully monitored treatment-associated reductions in bacterial load in one patient, observed both as an increase in Ct values in TB-MBLA and as prolonged TTP by MGIT culture. Previous studies reported approximately 70% agreement between TB-MBLA and TTP results (9, 24), indicating a degree of discrepancy between the two methods. In the PanACEA MAMS trial, which evaluated four experimental regimens, including higher-dose rifampicin, moxifloxacin, and SQ109 across five arms, TB-MBLA distinguished bacterial killing in four relapses versus 132 cured patients already at week 2, whereas TTP in MGIT detected significant differences only from week 8, showing the potential value of TB-MBLA (25). The main purpose of including the clinical samples in our study was to verify that the adapted TB-MBLA method also performed as expected in patient samples, in addition to inoculated sputum. Indeed, the very limited number of clinical samples in our study does not allow firm conclusions to be drawn regarding the correlation between TB-MBLA results obtained with the automated protocol and TTP, and remains to be investigated in larger clinical cohorts and multi-center studies. Additionally, the impact of sample quality from sample types other than sputum needs to be further investigated. Our main goal was to streamline the TB-MBLA protocol to simplify the workflow. However, as a molecular assay, even with automated extraction, it is still far from a fully automated point-of-care method and requires an advanced laboratory with specialized equipment and trained personnel. Broad implementation in high-burden, resource-limited settings would therefore require further development of the method into a point-of-care test.

## Supplementary Material

Reviewer comments

## SUPPLEMENTAL MATERIAL

Supplemental material spectrum.01018-26-s0001.docx Supplemental methods and results.

OPEN PEER REVIEW reviewer-comments.pdf An accounting of the reviewer comments and feedback.

ASM does not own the copyrights to Supplemental Material that may be linked to, or accessed through, an article. The authors have granted ASM a non-exclusive, world-wide license to publish the Supplemental Material files. Please contact the corresponding author directly for reuse.
