# Supplementary material for: Adaptation of the molecular bacterial load assay for real-time quantification of Mycobacterium tuberculosis in the clinical routine laboratory
Source: Microbiol Spectr. 2026 Aug 18;14(10):e01018-26. doi: 10.1128/spectrum.01018-26 (PMC13689229; doi:10.1128/spectrum.01018-26)
Supplement: Supplementary file 1 [file reviewer-comments.pdf]

ASM | Microbiology Spectrum

Adaptation of the molecular bacterial load assay (MBLA) for real-time quantification of Mycobacterium tuberculosis in the clinical routine laboratory

Blanka Andersson, Vita Kholod, Michaela Jonsson Nordvall, Clara Braian, Rajender Kumar, Katarina Niward, and Thomas Schön

Corresponding Author(s): Blanka Andersson, Linköping University

Review Timeline:

Submission Date:	March 23, 2026
Editorial Decision:	May 17, 2026
Revision Received:	July 8, 2026
Accepted:	July 20, 2026

Editor: Kayvan Zainabadi

Reviewer(s): Disclosure of reviewer identity is with reference to reviewer comments included in decision letter(s). The following individuals involved in review of your submission have agreed to reveal their identity: Abdulwahab Aliyu (Reviewer #1); Emmanuel Musisi (Reviewer #2)

Transaction Report:

(Note: With the exception of the correction of typographical or spelling errors that could be a source of ambiguity, letters and reports are not edited. The original formatting of letters and referee reports may not be reflected in this compilation.)

DOI: https://doi.org/10.1128/spectrum.01018-26

Re: Spectrum01018-26 (Adaptation of the molecular bacterial load assay (MBLA) for real-time quantification of Mycobacterium tuberculosis in the clinical routine laboratory)

Dear Dr. Blanka Andersson:

Thank you for the privilege of reviewing your work. Below you will find my comments, instructions from the Spectrum editorial office, and the reviewer comments.

I've had the manuscript reviewed by two experts in the field. Please note that Reviewer 2 refers to WHO 2018, not "2028".

In your revision, please also address these points:

1. Please provide a detailed protocol for the automated extraction method as a supplemental file.

2. Please provide more data confirming that 80C for 20min fully inactivates Mtb. What was the limit of detection of CFU? Why wasn't MGIT performed, which is more sensitive? Also equally important, how did the different inactivation methods affect MBLA Ct values?

2. Please provide more data on the purified RNA from both methods, including RNA/DNA concentrations, purity, and RNA integrity numbers if possible.

3. Please provide an explanation for this: "RNA obtained using the manual protocol required a 10 fold dilution to avoid inhibition in the RT qPCR reaction". Is this expected, ie was it observed/required in the original MBLA assay? If not, why is it required now?

4. What is the purpose of the sputolysine in treating non-TB sputum? Was TB sputum similarly treated with sputolysine? Does sputolysine contribute to Mtb inactivation with 80C?

5. 100 µl of RNA extraction control was added to inactivated sputum, but then sputum was centrifuged and only the pellet used. Shouldn't the RNA extraction control be lost in the discarded supernatant?

6. Please provide more info on the reproducibility of the automated protocol. For example, please provide the extraction control Ct values for the different sputum samples -- how much variability is there? Also if possible, please analyze more sputa, the small number used in the current study is a real weakness of this manuscript as sputa can vary widely between patients and thus provides little data on reproducibility for routine clinical use.

7. Please provide more detail on how your standard curve was created with presumably log-phase lab grown Mtb, and please comment how this relates to Mtb derived from patient sputum, both for patients before and during HRZE therapy.

8. Please provide more info on the patients used in this study.

9. "weeks to positive results" typo

Please return the manuscript within 60 days; if you cannot complete the modification within this time period, please contact me. If you do not wish to modify the manuscript and prefer to submit it to another journal, notify me immediately so that the manuscript may be formally withdrawn from consideration by Spectrum.

Revision Guidelines

To submit your modified manuscript, log into the submission site at https://spectrum.msubmit.net/cgi-bin/main.plex. Go to Author Tasks and click the appropriate manuscript title to begin. The information you entered when you first submitted the paper will be displayed; update this as necessary. Note the following requirements:

• Upload point-by-point responses to the issues raised by the reviewers in a file named "Response to Reviewers," NOT in your cover letter.
• Upload a compare copy of the manuscript (without figures) as a "Marked-Up Manuscript" file.
• Upload a clean .DOC/.DOCX version of the revised manuscript and remove the previous version.
• Each figure must be uploaded as a separate, editable, high-resolution file (TIFF or EPS preferred), and any multipanel figures must be assembled into one file.
• Any supplemental material intended for posting by ASM should be uploaded with their legends separate from the main manuscript. You can combine all supplemental material into one file (preferred) or split it into a maximum of 10 files with all associated legends included.

For complete guidelines on revision requirements, see our Submission and Review Process webpage. Submission of a paper that does not conform to guidelines may delay acceptance of your manuscript.

Data availability: ASM policy requires that data be available to the public upon online posting of the article, so please verify all links to sequence records, if present, and make sure that each number retrieves the full record of the data. If a new accession number is not linked or a link is broken, provide Spectrum production staff with the correct URL for the record. If the accession numbers for new data are not publicly accessible before the expected online posting of the article, publication may be delayed; please contact production staff (Spectrum@asmusa.org) immediately with the expected release date.

Publication Fees: For information on publication fees and which article types are subject to charges, visit ourwebsite. If your manuscript is accepted for publication and any fees apply, you will be contacted separately about payment during the production process; please follow the instructions in that e-mail. Arrangements for payment must be made before your article is published.

ASM Membership: Corresponding authors may join or renew ASM membership to obtain discounts on publication fees. Need to upgrade your membership level? Please contact Customer Service at Service@asmusa.org.

The ASM Journals program strives for constant improvement in our submission and publication process. Please tell us how we can improve your experience by taking this quick Author Survey.

Thank you for submitting your paper to Spectrum.

Sincerely,
Kayvan Zainabadi
Editor
Microbiology Spectrum

Reviewer #1 (Comments for the Author):

Their is need for improvement in some areas as highlighted in the reviewers comments.

Reviewer #2 (Comments for the Author):

TB-MBLA was recognized by the WHO in 2028 as a promising alternative to smear microscopy and culture for treatment monitoring, It quantifies viable Mycobacterium tuberculosis bacilli using ribosomal RNA within hours. It provides a dynamic assessment of bacterial clearance during treatment during therapy. Since RNA clears faster than DNA, RNA based assays are likely to give a more accurate complementary means of monitoring treatment response. However, in the current state, TB-MBLA has limitations including the need for a BSL3 lab, centrifugation and extensive hands-on time. These limitations may hinder its integration into routine care. The current study proposes important interventions which to narrow the list of limitations of this technology.

Abstract:
22-23: Authors state that "the current TB-MBLA protocol includes highly toxic chemicals......" what did they replace these chemicals with? I suggest that this they mention the less toxic chemicals that were used . I can see that the need for using a RNA preservative was eliminated and replaced by the lysis buffer. What are the components of the lysis buffer?
25-26: Heat inactivation was previously reported by Sabiiti et al., but I am interested in knowing the reliability of this approach for paucibacillary disease and other low burden samples. I see their approach eliminates centrifugation steps which is a good thing.
26-30: I am hesitant to refer to this protocol an automated one. It sounds like a semi-automated one. We still must lyse the samples outside the Maelstrom Switch 8 automated magnetic bead instrument. Still, we perfume DNAse digestion step outside the equipment- compared with Promega Maxwell.
MATERIAL AND METHOD
85-86: Preparation of sputum inoculated with Mtb; Well written
97-98: Evaluation of Mtb inactivation in sputum samples; well written
115-116: Sample preparation for TB-MBLA; Well written
129-130: Manual phenol-chloroform extraction of RNA; Well written
141-151: Automated extraction of RNA with Maelstrom Switch8; How much volume of the sample was collected? Was there any effect of sample: lysis buffer ratio? I know Invitrogen Turbo DNA-free{trade mark, serif} kit was used to digest off genomic DNA but it should be fine for the authors to mention some fine details like the sample: enzyme ratio, the incubation time and how they confirmed if DNAse digestion was successful.
152-166: Reverse transcription and quantitative PCR; well written
167-172: Conversion of Ct values to CFU/mL; well written. It is fine for the authors to cite the protocol
173-179: TTP determination: How was this sample obtained? Was a portion of what was extracted or were there two

independent samples collected from the same patient at different time points? Most likely one sample was collected (pooled sample) and apportioned to different assays.
184-189: How reproducible were the results? Did the authors have multiple experiments? It would be good to see how reproducible the results are.
Discussion: Different sample types, volumes, and quality may have different yields. This may need to be well discussed.

The Author have described an Adaptation of the molecular bacterial load assay (MBLA) for real-time quantification of *Mycobacterium tuberculosis* in the clinical routine laboratory aiming at rapid quantification of *Mycobacterium tuberculosis*. This work has demonstrated profound strength. However, the following need to be address:

1. The title in Figure 2 should be placed at the bottom of the figure not on top.
2. Although the author have demonstrated the linearity of the adopted method in line 401 under the supplementary method and result, there is need to determine the limit of detection (LoD) and limit of quantitation (LoQ) of the adopted method.
3. For the adopted method to be considered sound and valid, it has to show an acceptable percentage of recovery/detection (more than 70%) or in another way quantifies viable Mtb. In addition the method has to demonstrate lower RSDs for within-day and between-ady analysis to indicate good accuracy and precision of the method. The Arthor need to clearly demonstrate this while discussing their findings.

Review report: for the manuscript title "Adaptation of the molecular bacterial load assay (MBLA) for real-time quantification of 1 *Mycobacterium tuberculosis* in the clinical routine laboratory"

Summary of my review: dna removal time, how much was transferred,

TB-MBLA was recognized by the WHO in 2028 as a promising alternative to smear microscopy and culture for treatment monitoring, It quantifies viable Mycobacterium tuberculosis bacilli using ribosomal RNA within hours. It provides a dynamic assessment of bacterial clearance during treatment during therapy. Since RNA clears faster than DNA, RNA based assays are likely to give a more accurate complementary means of monitoring treatment response. However, in the current state, TB-MBLA has limitations including the need for a BSL3 lab, centrifugation and extensive hands-on time. These limitations may hinder its integration into routine care. The current study proposes important interventions which to narrow the list of limitations of this technology.

Abstract:

22-23: Authors state that "the current TB-MBLA protocol includes highly toxic chemicals……" what did they replace these chemicals with? I suggest that this they mention the less toxic chemicals that were used . I can see that the need for using a RNA preservative was eliminated and replaced by the lysis buffer. What are the components of the lysis buffer?

25-26: Heat inactivation was previously reported by Sabiiti et al., but I am interested in knowing the reliability of this approach for paucibacillary disease and other low burden samples. I see their approach eliminates centrifugation steps which is a good thing.

26-30: I am hesitant to refer to this protocol an automated one. It sounds like a semi-automated one. We still must lyse the samples outside the Maelstrom Switch-8 automated magnetic-bead instrument. Still, we perfume DNAse digestion step outside the equipment- compared with Promega Maxwell.

MATERIAL AND METHOD

85-86: Preparation of sputum inoculated with Mtb; Well written

97-98: Evaluation of Mtb inactivation in sputum samples; well written

115-116: Sample preparation for TB-MBLA; Well written

129-130: Manual phenol-chloroform extraction of RNA; Well written

141-151: Automated extraction of RNA with Maelstrom Switch8; How much volume of the sample was collected? Was there any effect of sample: lysis buffer ratio? I know Invitrogen Turbo DNA-free™ kit was used to digest off genomic DNA but it should be fine for the authors to mention some fine details like the sample: enzyme ratio, the incubation time and how they confirmed if DNAse digestion was successful.

152-166: **Reverse transcription and quantitative PCR; well written**

167-172: Conversion of Ct values to CFU/mL; well written. It is fine for the authors to cite the protocol

173-179: TTP determination: How was this sample obtained? Was a portion of what was extracted or were there two independent samples collected from the same patient at different time points? Most likely one sample was collected (pooled sample) and apportioned to different assays.

184-189: How reproducible were the results? Did the authors have multiple experiments? It would be good to see how reproducible the results are.

Discussion: Different sample types, volumes, and quality may have different yields. This may need to be well discussed.

Dear Editor, Professor Kayvan Zainabadi

We thank the reviewers and the Editorial team for valuable comments. By performing additional experiments, we have now added new data to our manuscripts to clarify and respond to the issues raised. We believe that this has improved the manuscript, and we are grateful for the input provided. Below, we have included a point-by-point reply to all comments. We hope that our manuscript is now suitable for publication in Microbiology Spectrum.

On behalf of the co-authors,

Blanka Andersson PhD

<u>Editorial comments</u>:

1. Please provide a detailed protocol for the automated extraction method as a supplemental file.

<u>Author response:</u>

We have used the suggested opportunity to include a detailed protocol for the automated extraction method in a supplemental method part. This supplement is now also referred to in the method section.

2. Please provide more data confirming that 80C for 20min fully inactivates Mtb. What was the limit of detection of CFU? Why wasn't MGIT performed, which is more sensitive? Also equally important, how did the different inactivation methods affect MBLA Ct values?

<u>Author response:</u>

1. According to the suggestion from the Editor, we performed new experiments investigating presence of growth in BACTEC 960 MGIT following inactivation of Mtb by 80 ℃ for 20min. We did not detect any growth in MGIT following heat inactivation. Indeed, MGIT is more sensitive (1-10 CFU) than LJ (10-100 CFU) and we discuss the LOD for both methods in the discussion part (page 18-19) where we also stress that any inactivation protocol must be carefully validated under local conditions and that samples should be handled in a BSL-2 biosafety cabinet since low level growth of Mtb has been reported by others by heat-inactivation and other methods. We initially used LJ medium alone as the inactivation control, as low-level growth (<10 CFU) cannot be entirely excluded even by MGIT culture due to variability in clinical sample conditions. However, any residual risk is mitigated using a BSL-2 cabinet, consistent with the procedure used for GeneXpert PCR sample preparation. In the present evaluation, sufficient inactivation was demonstrated using both MGIT and LJ culture. No growth was detected in MGIT following heat treatment at 80°C for 20 minutes, despite

inoculation with a high burden of M. tuberculosis (7×10^6 CFU) in sputum, whereas control cultures without heat inactivation exhibited rapid growth (positive after 5 days and 14 hours).

2. Regarding the effect of inactivation methods on MBLA Ct values, we selected heat-inactivation as we regarded this method the most resource effective to facilitate large-scale implementation. The main reason for the inactivation of *M. tuberculosis* is to be able to perform the whole procedure outside the BSL-3 laboratory and thus avoid putting all equipment needed for TB-MBLA in the BSL-3 laboratory which is beyond scope of most routine laboratories. Such implementation factors are now described in a more detailed way in the discussion part (page 18).

In our setting, it was not possible to investigate how inactivation methods affected TB-MBLA Ct values as the TB-MBLA assay could not be performed for non-inactivated samples outside the BSL-3 laboratory due to considerations of contagiousness. Still, we acknowledge the need for quantifying the effect of inactivation for the TB-MBLA method as raised by the Editor. Thus, to quantify the effect of inactivation, we used *M. bovis* BCG as a member and representative of the *M. tuberculosis* complex to enable analysis outside the BSL3-laboratory. We performed additional experiments to investigate how heating at 80C for 20 min affected TB-MBLA Ct values. In triplicate samples, there was no difference in mean TB-MBLA Ct-values between heat-inactivated samples (Ct 25.1) compared to control samples without inactivation (Ct 24.7), similar to the previously published results for TB-MBLA using the original extraction protocol (Sabiiti et al - reference 18 in manuscript). These results were included in the first part of the result section on page 11 and the results shown in Supplementary Table S1.

2. Please provide more data on the purified RNA from both methods, including RNA/DNA concentrations, purity, and RNA integrity numbers if possible.

Author response:

We have now provided RNA-concentration and purity from both automated and the original method respectively. This is now mentioned in the result section, page 11 and shown in Supplementary Table S2.

3. Please provide an explanation for this: "RNA obtained using the manual protocol required a 10 fold dilution to avoid inhibition in the RT qPCR reaction". Is this expected, ie was it observed/required in the original MBLA assay? If not, why is it required now?

Author response:

We have now included a more thorough explanation for the 10-fold dilution on page 19. In the original TB-MBLA protocol using phenol-chloroform extraction, a 10-fold dilution is recommended. Indeed, we also observed that the manual extraction did not generate amplification without the 10-fold dilution. In contrast, we did not observe a need for a 10-fold dilution using the automated protocol. We have now included these data in Supplementary Table S3.

4. What is the purpose of the sputolysine in treating non-TB sputum? Was TB sputum similarly treated with sputolysine? Does sputolysine contribute to Mtb inactivation with 80C?

Author response:

We agree that the purpose of adding sputolysine was not clear from the current method description which has now been clarified on page 6 as it is a part of the routine procedure in many diagnostic laboratories as well as in our setting. Sputolysine is added to all patient sputa which are submitted to the routine laboratory where it was collected. The purpose of sputolysine is that it is required for enabling work with sputum such as pipetting. For that reason, TB sputum is also always treated with sputolysine which is common in sputum preparation protocols for routine laboratories. In additional experiments, we found no effect on sputolysine at 80C for 20 min which showed similar bacterial loads to samples without sputolysine, expressed as time to positive culture (TTP) in MGIT (data shown below).

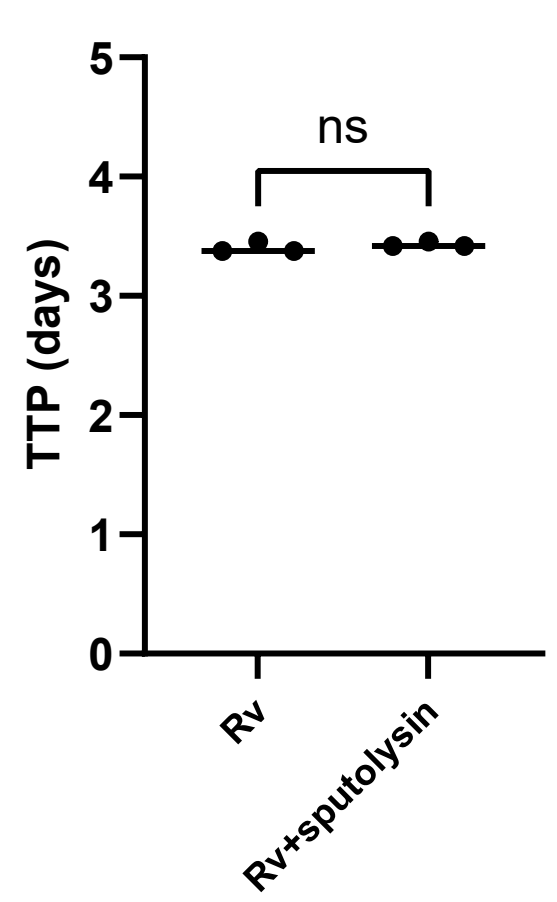

5. 100 µl of RNA extraction control was added to inactivated sputum, but then sputum was centrifuged and only the pellet used. Shouldn't the RNA extraction control be lost in the discarded supernatant?

Author response: The composition of the extraction control is different compared to pure RNA which would indeed have been lost in the discarded supernatant. However, the extraction control included from the TB-MBLA kit is composed of bacterial cells (not specified in the kit but most likely M. marinum as previously described by the authors), which are possible to pellet together with the sample and the RNA from the bacterial cells is extracted alongside the sample as shown by the extraction control now included in Figure 1. We have clarified the extraction control composition in the method section on the page 7 of the manuscript. We also rephrased "RNA extraction control" to the "extraction control" throughout the manuscript to clarify that it does not contain the pure RNA.

6. Please provide more info on the reproducibility of the automated protocol. For example, please provide the extraction control Ct values for the different sputum samples -- how much variability is there? Also if possible, please analyze more sputa, the small number used in the current study is a real weakness of this manuscript as sputa can vary widely between patients and thus provides little data on reproducibility for routine clinical use.

Author response:

According to the suggestion by the Editor, we have now included the extraction control Ct values in Figure 1. As shown in Figure 1, the variability is low and in general +/- 1 Ct value. We acknowledge the weakness of including few patient samples but in this work, we have put emphasis on the method development. Sputa may vary between patients, but the TB-MBLA method is indented for follow up during treatment for the same patient. Even so, we agree on the weakness of few patient samples, and we have now analysed some more patient samples, and we also emphasised the need for further multi-centre studies in the discussion part (page 20) which may be facilitated by our simplified protocol.

7. Please provide more detail on how your standard curve was created with presumably log-phase lab grown Mtb, and please comment how this relates to Mtb derived from patient sputum, both for patients before and during HRZE therapy.

Author response:

We have now described in more detail how the standard curve was created in the method section on page 6. It is always a challenge to evaluate how laboratory methods such as log-phase lab grown Mtb correlate to growth *in vivo*. In the case of TB-MBLA, the main clinical application would be to follow up the bacterial load dynamics in patients by comparing initial TB-MBLA levels with those after treatment rather than a single precise estimation of CFU levels. During the first weeks of TB treatment, rapidly growing bacteria can be detected by both TTP and TB-MBLA which also correlate to contagiousness and response to HRZE therapy

in previous studies which indicate that CFU levels from the standard curve derived from log-phase lab grown Mtb correlate well to the number of dividing bacteria in sputum *in vivo* quantified by TB-MBLA as 16S rRNA expression. Still, dormant subtypes are difficult to detect with any current method especially after weeks of HRZE therapy. These important aspects raised by the Editor were commented in the discussion section on page 19.

8. Please provide more info on the patients used in this study.

Author response: We have now included a table with baseline characteristics of patients included in the study as Table 1 on page 17.

9. "weeks to positive results" typo

Author response: Corrected.

Reviewer #1 (Comments for the Author):

There is need for improvement in some areas as highlighted in the reviewers comments. The Author have described an Adaptation of the molecular bacterial load assay (MBLA) for real-time quantification of Mycobacterium tuberculosis in the clinical routine laboratory aiming at rapid quantification of Mycobacterium tuberculosis. This work has demonstrated profound strength. However, the following need to be address:

1. The title in Figure 2 should be placed at the bottom of the figure not on top.

Author response: Corrected.

2. Although the author have demonstrated the linearity of the adopted method in line 401 under the supplementary method and result, there is need to determine the limit of detection (LoD) and limit of quantitation (LoQ) of the adopted method.

Author response: We have now included a determination of LoD for the automated method which is 100 CFU (Supplementary Table 5 and page 12 of the result section) whereas the LoQ was determined as 1000 CFU/ml where there was constant amplification at 100% of replicated samples.

3. For the adopted method to be considered sound and valid, it has to show an acceptable percentage of recovery/detection (more than 70%) or in another way quantifies viable Mtb. In addition the method has to demonstrate lower RSDs for within-day and between-day

analysis to indicate good accuracy and precision of the method. The Arthor need to clearly demonstrate this while discussing their findings.

Author response:

In accordance with evaluations of the original TB-MBLA method which also used inactivation at 80C for 20 min (Sabiiti et al - reference 18 in manuscript), we also verified that the method indeed quantifies viable Mtb which is plausible since our adjustment of the original TB-MBLA method concerns the automated extraction. The importance of recovery was stressed on page 18 in the discussion part. We also performed additional experiments showing similar TB-MBLA Ct levels with or without heat inactivation with a recovery of more than 95% which are now included as a Supplementary Table S1. To quantify the effect of inactivation, we used *M. bovis* BCG as a member and representative of the *M. tuberculosis* complex to enable analysis outside the BSL3-laboratory. In triplicate samples, there was no difference in mean TB-MBLA Ct-values between heat-inactivated samples (Ct 25.1) compared to control samples without heat inactivation (Ct 24.7). These results were included in the first part of the result section on page 11 and the results shown in Supplementary Table S1.

Reviewer #2 (Comments for the Author):
Summary of my review: dna removal time, how much was transferred,

TB-MBLA was recognized by the WHO in 2028 as a promising alternative to smear microscopy and culture for treatment monitoring, It quantifies viable Mycobacterium tuberculosis bacilli using ribosomal RNA within hours. It provides a dynamic assessment of bacterial clearance during treatment during therapy. Since RNA clears faster than DNA, RNA based assays are likely to give a more accurate complementary means of monitoring treatment response. However, in the current state, TB-MBLA has limitations including the need for a BSL3 lab, centrifugation and extensive hands-on time. These limitations may hinder its integration into routine care. The current study proposes important interventions which to narrow the list of limitations of this technology.

Abstract:
22-23: Authors state that "the current TB-MBLA protocol includes highly toxic chemicals......" what did they replace these chemicals with? I suggest that this they mention the less toxic chemicals that were used . I can see that the need for using a RNA preservative was eliminated and replaced by the lysis buffer. What are the components of the lysis buffer?

Author response: The components of the lysis buffer are guanidine salts, Tris buffer and surfactants. according to the manufacturer (TANBead® Nucleic Acid Extraction Kit, 6K2 series, Taiwan Advanced Nanotech, Taoyuan City, Taiwan). We clarified in the discussion section, page 17, that also other parts of the TB-MBLA method may contain toxic chemicals but not at the same rate as mercaptoethanol and phenol-chloroform.

25-26: Heat inactivation was previously reported by Sabiiti et al., but I am interested in knowing the reliability of this approach for paucibacillary disease and other low burden samples. I see their approach eliminates centrifugation steps which is a good thing.

Author response:

We selected heat inactivation as it is feasible also in high endemic settings. TB-MBLA can be used in most patients with paucibacillary disease down to a limit of detection at 100 CFU/ml which would correspond to very low to trace-levels in Xpert Ultra. The LOD has now been clarified on page 12 in the result section.

26-30: I am hesitant to refer to this protocol an automated one. It sounds like a semi-automated one. We still must lyse the samples outside the Maelstrom Switch 8 automated magnetic bead instrument. Still, we perfume DNAse digestion step outside the equipment-compared with Promega Maxwell.

Author response: We acknowledge this point by the reviewer and agree that it is not a fully automated method but rather an automated extraction procedure which is more automated than the cumbersome phenol-extraction method. A fully automated TB-MBLA method in a point-of-care format like Xpert Ultra will hopefully be developed by the diagnostic industry. We have included this issue in the discussion section, page 20.

MATERIAL AND METHOD
85-86: Preparation of sputum inoculated with Mtb; Well written

97-98: Evaluation of Mtb inactivation in sputum samples; well written
115-116: Sample preparation for TB-MBLA; Well written
129-130: Manual phenol-chloroform extraction of RNA; Well written

Author response: We thank the reviewer for the positive feedback.

141-151: Automated extraction of RNA with Maelstrom Switch8; How much volume of the sample was collected? Was there any effect of sample: lysis buffer ratio? I know Invitrogen Turbo DNA-free kit was used to digest off genomic DNA but it should be fine for the authors to mention some fine details like the sample: enzyme ratio, the incubation time and how they confirmed if DNAse digestion was successful.

Author response:

In the automated extraction, 250 µl of sputum was the maximum sample volume to load and the eluate was 60 µl. Since we found comparable results to the phenol-extraction method, we did not further optimize the extraction method and used a fixed sample:lysis buffer ratio

according to the instruction by the manufacturer. The volume of RNA prepared by the automated protocol from Mtb-inoculated sputum samples and TB patient samples entering DNAse treatment by Invitrogen Turbo DNA was 40µl. DNAse treatment was performed in two-step incubation, where 40µl of RNA was incubated with 1µl DNAse enzyme for 30 minutes and after that additional 1µl of DNAse enzyme was added and incubated for another 30 minutes.

We confirmed successful DNA digestion by testing the Mtb-inoculated sputum samples as we found no amplification below the cut off limit (Ct 37) from qPCR after DNAse treatment omitting the reverse transcriptase enzyme as shown by the data below.

qPCR without RT step

	**before DNAse treatment**		**after DNAse treatment**	
	**Mtb**	**EC**	**Mtb**	**EC**
$1x10^4$ A	Undetermined	27,904	Undetermined	Undetermined
$1x10^4$ B	36,324	27,743	Undetermined	Undetermined
$1x10^3$ A	36,046	25,656	Undetermined	Undetermined
$1x10^3$ B	38,551	28,219	37,285	Undetermined
$1x10^2$ A	35,912	27,343	37,014	Undetermined
$1x10^2$ B	37,242	25,180	Undetermined	Undetermined

152-166: Reverse transcription and quantitative PCR; well written
167-172: Conversion of Ct values to CFU/mL; well written. It is fine for the authors to cite the protocol

Author response: We thank the reviewer for the positive feedback.

173-179: TTP determination: How was this sample obtained? Was a portion of what was extracted or were there two independent samples collected from the same patient at different time points? Most likely one sample was collected (pooled sample) and apportioned to different assays.

Author response:

It was clarified that for comparison to TB-MBLA of clinical samples, the same sputum samples were divided and used for TTP and TB-MBLA analysis respectively.

184-189: How reproducible were the results? Did the authors have multiple experiments? It would be good to see how reproducible the results are.

Author response:

We have now included data on reproducibility including a Supplementary Table S5) for the data. The intra-assay variability (CV%) was 0.5% and the inter-assay variability 1.7 % which was well below the 10% target.

Discussion: Different sample types, volumes, and quality may have different yields. This may need to be well discussed.

Author response:

Samples types, volumes and sample quality are indeed important aspects, and we have included that in the discussion part on page 20 as an important limitation to be considered in future studies on the TB-MBLA method.

Re: Spectrum01018-26R1 (Adaptation of the molecular bacterial load assay (MBLA) for real-time quantification of Mycobacterium tuberculosis in the clinical routine laboratory)

Dear Dr. Blanka Andersson:

Your manuscript has been accepted, and I am forwarding it to the ASM production staff for publication. Your paper will first be checked to make sure all elements meet the technical requirements. ASM staff will contact you if anything needs to be revised before copyediting and production can begin. Otherwise, you will be notified when your proofs are ready to be viewed.

Data Availability: ASM policy requires that data be available to the public upon online posting of the article, so please verify all links to sequence records, if present, and make sure that each number retrieves the full record of the data. If a new accession number is not linked or a link is broken, provide production staff with the correct URL for the record. If the accession numbers for new data are not publicly accessible before the expected online posting of the article, publication may be delayed; please contact ASM production staff immediately with the expected release date.

Publication Fees: For information on publication fees and which article types have charges, please visit our website. We have partnered with Copyright Clearance Center (CCC) to collect author charges. If fees apply to your paper, you will receive a message from no-reply@copyright.com with further instructions. For questions related to paying charges through RightsLink, please contact CCC at ASM_Support@copyright.com or toll free at +1-877-622-5543. CCC makes every attempt to respond to all emails within 24 hours.

ASM Membership: Corresponding authors may join or renew ASM membership to obtain discounts on publication fees. Need to upgrade your membership level? Please contact Customer Service at Service@asmusa.org.

Embargo Policy: A press release may be issued as soon as the manuscript is posted on the Spectrum Latest Articles webpage. The corresponding author will receive an email with the subject line "ASM Journals Author Services Notification" when the article is available online.

Cover Image Submissions: If you would like to submit a potential Cover Image, please email a file and a short legend to spectrum@asmusa.org. Please note that we can only consider images that (i) the authors created or own and (ii) have not been previously published. By submitting, you agree that the image can be used under the same terms as the published article. Image File requirements: TIF/EPS, 7.5 inches wide by 8.25 inches tall (at least 2,250 pixels wide by 2,475 pixels tall), minimum 300 dpi resolution (600 dpi preferred), RGB, and no figure elements, e.g., arrows or panel labels. The legend should be a short description of the image, 1-2 sentences recommended. Please download and use this interactive template in Adobe the https://journals.asm.org/pb-assets/html/asm-cover-sizing-tool.html to ensure that your proposed cover image meets our size requirements (https://journals.asm.org/pb-assets/pdf-text-excel-files/ASM-Interactive-Sizing-Cover-Template-1715689791.pdf).

The ASM Journals program strives for constant improvement in our submission and publication process. Please tell us how we can improve your experience by taking this quick Author Survey.

Thank you for submitting your paper to Spectrum.

Sincerely,
Kayvan Zainabadi
Editor
Microbiology Spectrum

Reviewer #1 (Comments for the Author):

The author have revised the manuscript as requested.

Reviewer #2 (Comments for the Author):

I have read the revised version and it is in a better shape than the when it was before the revision. The supplementary material add another layer of information, which is good. The responses to reviewer comments are sufficient.

Review report: for the manuscript title “Adaptation of the molecular bacterial load assay (MBLA) for real-time quantification of Mycobacterium tuberculosis in the clinical routine laboratory”

Abstract:
22-23: Authors state that "the current TB-MBLA protocol includes highly toxic chemicals......" what did they replace these chemicals with? I suggest that this they mention the less toxic chemicals that were used . I can see that the need for using a RNA preservative was eliminated and replaced by the lysis buffer. What are the components of the lysis buffer?

Author response: The components of the lysis buffer are guanidine salts, Tris buffer and surfactants. according to the manufacturer (TANBead® Nucleic Acid Extraction Kit, 6K2 series, Taiwan Advanced Nanotech, Taoyuan City, Taiwan). We clarified in the discussion section, page 17, that also other parts of the TB-MBLA method may contain toxic chemicals but not at the same rate as mercaptoethanol and phenol-chloroform.

I can confirm that this is now clarified in the discussion section.

25-26: Heat inactivation was previously reported by Sabiiti et al., but I am interested in knowing the reliability of this approach for paucibacillary disease and other low burden samples. I see their approach eliminates centrifugation steps which is a good thing.

Author response:

We selected heat inactivation as it is feasible also in high endemic settings. TB-MBLA can be used in most patients with paucibacillary disease down to a limit of detection at 100 CFU/ml which would correspond to very low to trace-levels in Xpert Ultra. The LOD has now been clarified on page 12 in the result section.

Ok. It is good that there is data on the Limit of detection on page 12 in the result section.

26-30: I am hesitant to refer to this protocol an automated one. It sounds like a semi-automated one. We still must lyse the samples outside the Maelstrom Switch 8 automated magnetic bead instrument. Still, we perfume DNAse digestion step outside the equipment- compared with Promega Maxwell.

Author response: We acknowledge this point by the reviewer and agree that it is not a fully automated method but rather an automated extraction procedure which is more automated than the cumbersome phenol-extraction method. A fully automated TB-MBLA method in a point-of-care format like Xpert Ultra will hopefully be developed by the diagnostic industry. We have included this issue in the discussion section, page 20.

Ok. It satisfying that this point is noted on page 20 of the revised version.

MATERIAL AND METHOD
85-86: Preparation of sputum inoculated with Mtb; Well written

97-98: Evaluation of Mtb inactivation in sputum samples; well written
115-116: Sample preparation for TB-MBLA; Well written
129-130: Manual phenol-chloroform extraction of RNA; Well written

Author response: We thank the reviewer for the positive feedback.

141-151: Automated extraction of RNA with Maelstrom Switch8; How much volume of the sample was collected? Was there any effect of sample: lysis buffer ratio? I know Invitrogen Turbo DNA-free kit was used to digest off genomic DNA but it should be fine for the authors to mention some fine details like the sample: enzyme ratio, the incubation time and how they confirmed if DNAse digestion was successful.

Author response:

In the automated extraction, 250 µl of sputum was the maximum sample volume to load and the eluate was 60 µl. Since we found comparable results to the phenol-extraction method, we did not further optimize the extraction method and used a fixed sample:lysis buffer ratio according to the instruction by the manufacturer. The volume of RNA prepared by the automated protocol from Mtb-inoculated sputum samples and TB patient samples entering DNAse treatment by Invitrogen Turbo DNA was 40µl. DNAse treatment was performed in two-step incubation, where 40µl of RNA was incubated with 1µl DNAse enzyme for 30 minutes and after that additional 1µl of DNAse enzyme was added and incubated for another 30 minutes.

We confirmed successful DNA digestion by testing the Mtb-inoculated sputum samples as we found no amplification below the cut off limit (Ct 37) from qPCR after DNAse treatment omitting the reverse transcriptase enzyme as shown by the data below.

qPCR without RT step

	**before DNAse treatment**		**after DNAse treatment**	
	**Mtb**	**EC**	**Mtb**	**EC**
$1x10^4$ A	Undetermined	27,904	Undetermined	Undetermined
$1x10^4$ B	36,324	27,743	Undetermined	Undetermined
$1x10^3$ A	36,046	25,656	Undetermined	Undetermined
$1x10^3$ B	38,551	28,219	37,285	Undetermined
$1x10^2$ A	35,912	27,343	37,014	Undetermined
$1x10^2$ B	37,242	25,180	Undetermined	Undetermined

I am ok with the response

152-166: Reverse transcription and quantitative PCR; well written
167-172: Conversion of Ct values to CFU/mL; well written. It is fine for the authors to cite the protocol

Author response: We thank the reviewer for the positive feedback.

173-179: TTP determination: How was this sample obtained? Was a portion of what was extracted or were there two independent samples collected from the same patient at different time points? Most likely one sample was collected (pooled sample) and apportioned to different assays.

Author response:

It was clarified that for comparison to TB-MBLA of clinical samples, the same sputum samples were divided and used for TTP and TB-MBLA analysis respectively.

Ok

184-189: How reproducible were the results? Did the authors have multiple experiments? It would be good to see how reproducible the results are.

Author response:

We have now included data on reproducibility including a Supplementary Table S5) for the data. The intra-assay variability (CV%) was 0.5% and the inter-assay variability 1.7 % which was well below the 10% target.

I am happy the revised version includes data on reproducibility with such a good inter and intra assay variability.

Discussion: Different sample types, volumes, and quality may have different yields. This may need to be well discussed.

Author response:

Samples types, volumes and sample quality are indeed important aspects, and we have included that in the discussion part on page 20 as an important limitation to be considered in future studies on the TB-MBLA method.

Ok to cite this as a limitation.
