# Supplementary material for: Adaptation of the molecular bacterial load assay for real-time quantification of Mycobacterium tuberculosis in the clinical routine laboratory
Source: Microbiol Spectr. 2026 Aug 18;14(10):e01018-26. doi: 10.1128/spectrum.01018-26 (PMC13689229; doi:10.1128/spectrum.01018-26)
Supplement: Supplementary file 2 [file spectrum.01018-26-s0001.docx]

**Supplementary methods**

**Preparation of sputum inoculated with *M. avium* and *M. bovis* BCG**

*Mycobacterium avium* strain ATCC 700898 and *M.bovis* BCG were thawed and cultured in Middlebrook 7H9 broth supplemented with Tween-80 and 10% OADC or ADC respectively (Becton Dickinson, Franklin Lakes, NJ, USA) for 2 to 3 weeks at 37°C. Bacterial cultures were reinoculated into fresh broth 7 days prior to the use in the experiments. Single-cell suspension of *Mycobacterium avium* or *M. bovis* BCG was prepared by passing the suspension 10x through 27-gauge needle both in the last wash and in PBS. The concentration of bacterial suspension in PBS was determined by optical density at 600 nm as a function of CFU/mL.

**Automated protocol with Qiagen EZ2**

Samples were heat inactivated at 80 °C for 20 min. The RNA was extracted using EZ1&2 RNA Tissue Mini Kit (Qiagen, Hilden, Germany). Samples together with 100 µl of the extraction control were lysed and homogenized in 600 µl RLT buffer containing 2% of 2M DTT. To be able to retrieve sufficient volume of lysate, since the extensive foaming during the homogenization procedure, the volume of lysing buffer was doubled from 300 µl to 600 µl. A total of 300 µl lysate was loaded together with 10 µl of reconstituted DNAse enzyme (Qiagen, Hilden, Germany) supplied with the kit and RNA extraction was performed using the EZ2®Connect instrument (Qiagen, Hilden, Germany) using Total RNA from Easy-to-Lyse Tissues program. The choice of program was based on the weight of pellet obtained from 250 µl sputum, which was around 10 mg.

**Reverse transcription and quantitative PCR**

Reverse transcription and quantitative PCR were performed as one-step reaction using MAC MBLA kit as recommended by the manufacturer (University of St Andrews, Fife, UK). As for the TB-MBLA kit, reaction components for both RT^+^ and RT^-^ reactions were pre-mixed and stored at -20 °C. Reaction components for both RT+ and RT- master mix with/out reverse transcriptase enzyme respectively were pre-mixed after kit arrival and stored at -20°C. One reaction contained 4 µl of sample and 16 µl of master mix. Samples were tested in duplicates in RT^+^ reaction and single reaction in RT^-^ reaction on Microamp Fast Optical 96-well plate in QuantStudio™ 7 Flex system (Applied Biosystems, Thermofisher, Waltham, MA, USA) using FAM and VIC channel for MAC 16S rRNA and extraction control, respectively. Inoculated-in extraction controls were used for normalization to compensate for the variation in the RNA recovery. Statistical differences were calculated using two-way Anova with Šidák´s multiple comparisons test in GraphPad Prism version 10.0.2. Data are presented as a mean ± SD and p-value <0.05 considered as significant.

**Supplementary laboratory method protocol for the TB-MBLA including automated extraction.**

**The TB-MBLA was performed based on the original TB-MBLA protocol with heat-inactivation (1) and adaptation for the automated extraction.**

**Sample preparation and heat inactivation**

- For heat-inactivation, carefully transfer 250 µl of sputum to a 1.5 ml sterile screw cap microtube (Sarstedt, Nümbrecht, Germany) inside a class II laminar flow hood (according to the local biosafety regulations).
- Inside the laminar flow hood/BSL-3 laboratory, load tubes onto foam-rubber rack and heat-inactivate, submerged in 80 °C waterbath, for 20 min. The temperature in the water bath should be carefully controlled by an external thermometer. (NB: the inactivation procedure should be validated several times in a BSL3 facility to make sure that no viable Mtb are present using the procedure with the local equipment).
- Allow samples to cool down for at least 5 min.
- After this step, sputum samples containing Mtb can be handled as non-contagious outside the BSL3 laboratory (but local biosafety regulations need to be followed).
- To each sputum sample, add 100 µl of extraction control (Vitalbacteria, St Andrews, Fife, UK).
- Centrifuge samples at 20,000 x g for 10 min and discard supernatant leaving 25 µl to keep pellet intact.
- Resuspended pellet in 800µl lysis buffer (TANBead® Nucleic Acid Extraction Kit, 6K2 series, Taiwan Advanced Nanotech, Taoyuan City, Taiwan), transfer to Lysing Matrix B tubes containing 0.1-mm silica beads (MP Biomedicals, Irvine, CA, USA) and homogenize using a BeadBug Mini homogenizer D1030 (Benchmark, Sayreville, NJ, USA) at maximum speed for two bursts of 40 s cycles separated by a 40 s rest.
- Homogenized samples in Lysing Matrix B tubes can be stored at -80 °C for a maximum of 2 weeks (see validation in manuscript, Fig 3) or processed immediately for RNA extraction.

**Automated extraction**

- Combine equal volumes of lysate and 99,5 % molecular biology grade isopropanol (Thermoscientific, Thermofisher, Waltham, MA, USA) (500 µL each) and process for RNA extraction on the Maelstrom Switch‑8 automated magnetic‑bead instrument (TANBead, Taiwan Advanced Nanotech, Taoyuan City, Taiwan) using the SW8-6K2 program resulting in a 60 µl eluate.
- Directly after extraction, take an aliquot of RNA eluate (40 µl) and subject to DNAse treatment using Invitrogen Turbo DNA-free™ kit (Invitrogen, Thermofisher, Waltham, MA, USA) according to the manufacturer instructions.

**Reverse transcription and quantitative PCR**

- Perform One‑step RT‑qPCR using Vitalbacteria TB-MBLA kit as recommended by the manufacturer (Vitalbacteria, St Andrews, Fife, UK). Reaction components for both the RT^+^ and RT^-^ master mix, with and without reverse transcriptase enzyme respectively, can be pre-mixed after kit arrival and stored at –20 °C.
- One reaction contains 4 µl of sample and 16 µl of master mix. Analyze samples in duplicates in RT^+^ reaction and in single reaction in RT^-^ reaction on Microamp Fast Optical 96-well plate in QuantStudio™ 7 Flex system (Applied Biosystems, Thermofisher, Waltham, MA, USA) using FAM and VIC channel for Mtb 16S rRNA and extraction control respectively.

**Supplementary results**

**Optimization of automated extraction protocol**

To automate and standardize RNA extraction protocol and to further decrease the use of toxic chemicals such as phenol and chloroform, we have compared the automated protocols using Maelstrom Switch8 extraction robot (TANBead) and EZ2 Connect extraction robot (Qiagen). Both protocols were tested using *M. avium* suspension in a 10-fold range from 1 × 10^6^ – 1 × 10^4^ CFU/mL of negative sputum in duplicates in two independent experiments. Input volume of sputum was 250 µl in both protocols. Mean Ct values for tested concentrations were significantly lower for samples extracted by Maelstrom Switch8 compared to mean Ct values for samples extracted by Qiagen protocol (Fig. S1).

**Fig. S1.** Comparison of Qiagen and Maelstrom automated RNA extraction protocols. Sputum samples inoculated with 10-fold dilution series of MAC suspension were heat-inactivated at 80 °C for 20 min and an extraction control (100 µl/sample) was added to all samples. MAC was quantified by MAC MBLA RT-qPCR following the automated RNA extraction protocol. Data are presented as mean Ct values ±SD (n=4) and difference shown as **** (p<0.0001).

**Fig S2.** Correlation between concentration of Mtb-inoculated sputum samples in CFU/mL and Ct values for samples determined by TB-MBLA RT-qPCR. The concentration of the Mtb suspension was estimated from the OD measurements, diluted 100-fold, and inoculated to the sputum samples in duplicates. TB-MBLA was performed using an adapted TB-MBLA protocol with automated RNA extraction. For CFU quantification, the standard curve should be repeated when a new kit of TB-MBLA is initiated. The standard curve was generated using linear regression. The correlation between Ct and CFU/mL sputum is expressed as R^2^.

| BCG+sputum | Ct | Range |
| --- | --- | --- |
| not inactivated | 24.7 | (24.5-24.8) |
| 80 ℃, 20 min | 25.1 | (25.1-25.1) |

**Table S1.** The impact of heat inactivation on TB-MBLA Ct values using the automated extraction protocol. Mean Ct-values and range of samples processed in triplicates is shown for *M. bovis* BCG (2.5x10^5^/sample) as a representative of the *M. tuberculosis* complex.

| Cfu/sample | Extraction protocol | RNA concentration (ng/µl) | RNA purity  260/280 |
| --- | --- | --- | --- |
| 2.5x10^5^ | Automated | 8.2 | 1.39 |
| 2.5x10^5^ | Automated | 7.5 | 1.43 |
| 2.5x10^2^ | Automated | 7.7 | 1.44 |
| 2.5x10^2^ | Automated | 5.9 | 1.22 |
| 1x10^6^ | Manual | 6.2* | 1.55 |
| 1x10^6^ | Manual | 9.7* | 1.62 |
| 1x10^3^ | Manual | 5.6* | 1.63 |
| 1x10^3^ | Manual | 7.1* | 1.59 |

**Table S2.** RNA concentration and purity in the automated compared to the manual phenol-chloroform extraction protocol for TB-MBLA analysed with Nanodrop. * The RNA concentration was adjusted for 4 times higher starting sputum volume of Mtb-inoculated sputum entering the phenol chloroform extraction (1ml) compared to the automated protocol (250µl).

|  | Automated extraction protocol | | | | | Manual extraction protocol | | | | |
| --- | --- | --- | --- | --- | --- | --- | --- | --- | --- | --- |
|  | Undiluted RNA | | 10x diluted RNA | | Undiluted RNA | | | 10x diluted RNA | |  |
| CFU/ml sputum | Mtb Ct | EC  Ct | Mtb  Ct | EC  Ct | Mtb  Ct | | EC  Ct | Mtb  Ct | EC  Ct |  |
| 1x10^4^A | 30.3 | 21.9 | 30.7 | 25.0 | 29.1 | | 28.8 | 31.1 | 29.8 |  |
| 1x10^4^A | 30.2 | 22.1 | 30.4 | 25.0 | 27.0 | | 26.5 | 30.9 | 29.8 |  |
| 1x10^4^B | 30.9 | 22.1 | 30.8 | 25.5 | ND | | ND | 29.6 | 27.8 |  |
| 1x10^4^B | 30.5 | 22.0 | 30.5 | 25.4 | 36.7 | | ND | 29.8 | 27.6 |  |
| 1x10^3^A | 33.9 | 21.5 | 33.2 | 24.0 | 39.7 | | ND | 32.2 | 29.2 |  |
| 1x10^3^A | 32.1 | 20.3 | 33.1 | 23.9 | ND | | ND | 32.7 | 29.4 |  |
| 1x10^3^B | 32.8 | 21.9 | 33.5 | 25.4 | ND | | ND | 31.9 | 29.1 |  |
| 1x10^3^B | 33.8 | 22.1 | 34.4 | 25.4 | ND | | ND | 31.5 | 29.4 |  |

**Table S3.** The impact of a 10-fold dilution of RNA-extracts in the manual and automated extraction protocol respectively. Undiluted samples in the automated extraction protocol did not always show positive results for Mtb or EC in contrast to samples diluted 10-fold. In contrast, there were no differences between undiluted and 10-fold diluted samples when the automated extraction protocol was applied. ND indicates no Ct value detected in qPCR reaction.

| CFU/ml sputum | Ct±SD (automated Switch8 Maelstrom protocol) | Ct±SD (manual phenol-chloroform protocol) |
| --- | --- | --- |
| 1x10^4^ | 31.6±1.0 | 30.9*±1.8 |
| 1x10^3^ | 34.3±0.7 | 33.2*±2.8 |
| 1x10^2^ | 36.0±1.4 | 35.5*±3.6 |

**Table S4.** Comparison of manual phenol-chloroform and an automated magnetic bead-based RNA extraction protocol. Mean Ct values and standard deviation (SD) for data presented in Fig. 1. *Ct values were adjusted for 4x larger starting sputum volume of Mtb inoculated sputum entering the phenol-chloroform extraction (1ml) compared to the automated protocol (250µl).

|  | Ct | | | | |
| --- | --- | --- | --- | --- | --- |
| CFU/ml sputum | Exp1 | Exp2 | Exp3 | Exp4 |  |
| 1x10^4^ | 31.5 | 32.6 | 30.9 | 31.4 |  |
|  | 31.5 | 32.5 | 31.0 | 31.2 |  |
|  | 31.7 |  |  |  |  |
|  | 31.6 |  |  |  |  |
|  | 31.9 |  |  |  |  |
|  | 31.6 |  |  |  |  |
| 1x10^3^ | 35.0 |  |  |  |  |
|  | 34.7 |  |  |  |  |
|  | 35.3 |  |  |  |  |
| 1x10^2^ | 37.3 |  |  |  |  |
|  | 38.0 |  |  |  |  |
|  | ND |  |  |  |  |
| 1x10^1^ | 37.0 |  |  |  |  |
|  | 37.3 |  |  |  |  |
|  | ND |  |  |  |  |

**Table S5.** Determination of Limit of detection (LOD) by replicated (n=3-6) dilutions of *M. tuberculosis*. ND indicates no Ct value detected in qPCR reaction.

**REFERENCES**

1.	Sabiiti W, Mtafya B, De Lima DA, Dombay E, Baron VO, Azam K, et al. A Tuberculosis Molecular Bacterial Load Assay (TB-MBLA). J Vis Exp. 2020(158).
